# Brain SPECT perfusion and PET metabolism as discordant biomarkers in major depressive disorder

**DOI:** 10.1186/s13550-020-00713-2

**Published:** 2020-10-08

**Authors:** Maud Tastevin, Laurent Boyer, Theo Korchia, Guillaume Fond, Christophe Lançon, Raphaëlle Richieri, Eric Guedj

**Affiliations:** 1 Department of Psychiatry, Sainte Marguerite University Hospital, Assistance Publique- Hôpitaux de Marseille, Marseille, France; 2 CEReSS-Health Service Research and Quality of Life Centre, Aix Marseille University, Marseille, France; 3 Department of Medical Information and Public Health, APHM, Marseille, France; 4 Department of Epidemiology and Health Economics, Assistance Publique-Hôpitaux de Marseille, Marseille, France; 5 CNRS, Centrale Marseille, Institut Fresnel, Aix Marseille University, Marseille, France; 6 Nuclear Medicine Department, APHM, CNRS, Centrale Marseille, Institut Fresnel, Timone Hospital, CERIMED, Aix Marseille University, Marseille, France

**Keywords:** Treatment-resistant depression, Major depressive disorder, PET, SPECT, Biomarker

## Abstract

**Background:**

Brain SPECT perfusion and PET metabolism have been, most often interchangeably, proposed to study the underlying pathological process in major depressive disorder (MDD). The objective of this study was to specify similarities and inconsistencies between these two biomarkers according to global characteristics of the disease. We conducted a retrospective study in 16 patients suffering from treatment-resistant MDD who underwent, during the same current episode, a cerebral perfusion SPECT with ^99m^Tc-HMPAO and a metabolic PET with ^18^F-FDG. Whole-brain voxel-based SPM(T) maps were generated in correlation with the number of depressive episodes and in correlation with the depression duration, separately for the two exams (p-voxel < 0.001 uncorrected, *k* > 20).

**Results:**

No significant correlations were found between brain metabolism and either the number of depressive episodes or the duration of the disease, even at an uncorrected p-voxel < 0.005. On the other hand, the increased number of depressive episodes was correlated with decreased perfusion of the right middle frontal cortex, the right anterior cingulum cortex, the right insula, the right medial temporal cortex and the left precuneus. The increased depression duration was correlated with decreased perfusion of the right anterior cingulum cortex.

**Conclusions:**

This preliminary study demonstrates more significant results with brain perfusion compared with glucose metabolism in treatment-resistant MDD, highlighting the value of brain SPECT despite less favourable instrumentation detection compared to PET.

## Background

Major depressive disorder (MDD) is a common mental health disorder. It is now the first cause of disability worldwide and a major contributor to the overall global burden of diseases according to the World Health Organization. It has been estimated that 15–30% of patients present with treatment-resistant depression (TRD), which is resistant to antidepressants and cognitive behavioural treatment [1].

The current diagnosis and course evaluation of MDD rely on clinical examination. To date, the risk of misdiagnosis remains present because of the lack of non-invasive and quantifiable assessments of all depression dimensions [2]. Several promising treatments have been proposed (pharmacological, non-invasive and invasive neuromodulation) in the case of antidepressant resistance. Nonetheless, the pathophysiology of depression and the neural and biological mechanisms of treatment efficacy are still not fully understood. In this context, neuroimaging biomarkers are needed for diagnosing, predicting the course of the disorder and guiding the choice of therapy, as well as monitoring the response to these therapies.

Since 2008, with the development of the “Rdoc Research” programme, brain SPECT with ^99m^Tc-ECD or ^99m^Tc-HMPAo and ^18^F-FDG PET have been proposed to, respectively, study regional blood flow and glucose metabolism in a range of psychiatric disorders, including MDD [3]. Despite distinct underlying mechanisms (perfusion vs. metabolism), both biomarkers are supposed to be coupled with the global synaptic activity [4]. They have been used to help in the neurological differential diagnosis of depression, and more recently considered to better understand the underlying pathological process in MDD and to predict response and non-response outcomes to neuromodulation therapies, such as repetitive transcranial magnetic stimulation (rTMS) or deep brain stimulation (DBS) [5, 6]. In this line, some brain regions seem particularly involved (e.g. the frontal cortex and more broadly the limbic system), especially in treatment-resistant cohorts. Nevertheless, the genuine overlap across PET/SPECT studies is globally more limited and slows down the clinical integration of these biomarkers into the patient’s evaluation. The main explanations of these discrepancies are the small size and clinical heterogeneity of inclusions, as well as the variability of neuroimaging techniques, radiotracers and statistical models [7]. One alternative hypothesis would be that neuroimaging biomarkers are not as equivalent as anticipated in MDD, especially for perfusion and metabolism.

The objective of this study was to specify similarities and inconsistencies between brain perfusion and brain glucose metabolism according to the global characteristics of the disease (i.e. the number of previous depressive episodes and the depression duration) in a single group of patients with MDD.

## Methods

### Subjects

We conducted a retrospective study. The database included patients with psychiatric follow-up at Sainte Marguerite University Hospital (Marseille, France) from January 2011 through July 2019. Our inclusion criteria were patients over the age of 18 suffering from TRD who underwent a cerebral ^99m^Tc-HMPAo SPECT and a ^18^F-FDG PET with an interval delay of less than 18 months during a same current major depressive episode (according to DSM-IV criteria). These SPECT examinations had been performed to initially explore differential diagnoses [8]. The selected patients were subsequently included in the HrTMS trial, which included a metabolic PET imaging evaluation before treatment with ethical and regulatory authorizations (ClinicalTrials.gov: NCT02559466; Registry Identifier ID RCB: 2015-A00345-44) [6, 9]. Patients with bipolar depression, schizophrenia or neurological comorbidities were excluded.

### Data collection

The sociodemographic characteristics recorded included gender, age, marital status and education level. Clinical data included illness duration, number of depressive episodes, and melancholic and psychotic characteristics, as well as pharmaco-resistance and global severity according to the DSM-IV (SCID-IV) structured clinical interview [10]. Data concerning severity scales, such as the MADRS or Beck, were not available in all patients for the two evaluation time points. Treatment data recorded included all antidepressants, antipsychotics and mood stabilizers, as well as invasive and non-invasive brain stimulation (electroconvulsive therapy, rTMS and deep brain stimulation and vagal nerve stimulation).

### SPECT and PET acquisitions

SPECT and PET scans were performed for all subjects, with the same SPECT and PET cameras, and under the same conditions. PET was performed using an integrated PET/CT camera (Discovery ST, GE Healthcare, Waukesha, WI, USA). Patients were required to fast for at least 6 h before undergoing the scan, with a control of normal blood glucose level. They were maintained in neurosensory resting 10 min before and 30 min after injection. ^18^F-FDG was injected intravenously at the activity of 150 MBq, and PET images were then acquired over a period of 15 min. Iterative reconstruction was performed on a matrix of 192 × 192, with correction of attenuation using CT acquisition. SPECT acquisition was performed using a double-headed rotating gamma camera (ECAM, Siemens) equipped with a fan beam collimator. Patients were maintained in neurosensory resting 10 min before and 20 min after intravenous injection of 740 MBq of ^99m^Tc-HMPAO. The total scan time was of 25 min with sixty projections per head of 25 s, collected in 128 × 128 format. Tomographic 3D reconstruction was performed using a filtered back-projection algorithm.

Images were initially converted from the DICOM to the NifTi format using MRIcro (www.mricro.com) and then transferred to SPM. Whole-brain statistical analysis was performed at the voxel level using SPM8 software (Wellcome Department of Cognitive Neurology, University College, London, UK) after spatial normalization (the Montreal Neurological Institute atlas) and smoothing with a Gaussian filter (8 mm full-width at half-maximum) to blur individual variations in gyral anatomy and to increase the signal-to-noise ratio. Whole-brain voxel-based SPM(T) maps were generated in correlation with the number of depressive episodes and in correlation with the depression duration, separately for the two exams (p-voxel < 0.001 uncorrected, *k* > 20), using proportional scaling with a grand mean scaled value fixed at 50.

### Statistical analysis

The Shapiro–Wilk test was used to confirm the normality of the two variables tested at PET and SPECT time examination [i.e. the depression duration (*p* = 0.017 and *p* = 0.011, respectively) and the number of episodes (*p* < 0.001)]. Data were presented in proportions or means and standard deviations. Characteristics were compared for each patient between PET and SPECT acquisition dates using Student’s t test or the Mann–Whitney *U* test for continuous variables and the chi-square test or Fisher’s exact test for categorical variables. Mean PET/SPECT values were extracted using MARSBAR software (https://marsbar.sourceforge.net/) at the individual level for significant cluster(s) to calculate Spearman’s correlations. The statistical significance level was set at *p* < 0.05 in a two-sided test.

## Results

### Baseline characteristics

A total of 16 subjects were included in the study. Patient characteristics are described in Table 1. Patients were mostly women with a mean age of approximately 50 years old. Our sample included 4 men and 12 women; 13 patients were single, and 10 had less than 12 years of education. They all presented with TRD with a mean number of depressive episodes estimated at 2.31 ± 1.9. Among this group, 13 patients suffered from a severe current episode, and melancholic criteria were present in 6 patients. The mean interval delay between PET and SPECT acquisitions was 4.37 ± 4.89 months (20 days to 13 months). No significant changes were found between these two time point evaluations, especially for clinical characteristics and treatments. Moreover, no treatment interruption occurred during the interval delay. However, age and disease duration did show statistically significant differences, with a respective difference of 5.28 months (*p* = 0.02) and 1.44 months (*p* = 0.023), respectively; these limited but significant differences were expected, since SPECT was systematically performed before PET in these patients.

**Table 1:** Demographic and clinical characteristics of patients at PET and SPECT day acquisitions (*n* = 16)

	At PET day acquisition	At SPECT day acquisition	*p* value
Demographic characteristics			
Age (years, mean ± SD)	50.50 ± 16.3	50.06 ± 16.7	**0.020**
Education			1.000
> 12 years	5	5	1.000
≤ 12 years	10	10	
Marital status			1.000
Single	13	13	1.000
Couple	3	3	
Clinical characteristics			
Depression duration (months)	136.44 ± 120	132.56 ± 121.48	**0.023**
Number of depressive episodes	2.31 ± 1.9	2.31 ± 1.9	1.000
SCID severity			
Mild	2	2	1.000
Moderate	1	1	1.000
Severe	13	13	1.000
Melancholic features	6	6	1.000
Treatments			
SSRIs	5	7	0.500
SNRIs	6	5	0.300
Tricyclics	2	2	1.000
MAOIs	1	1	1.000
Pramipexole	1	1	1.000
Antipsychotics, first generation	1	1	1.000
Antipsychotics, second generation	3	4	0.607
Mood stabilizers	0	0	1.000
Others	3	2	0.350

Significant differences are in bold

*SCID* structured clinical interview for DSM-IV, *SSRI* selective serotonin reuptake inhibitor, *SNRI* serotonin–norepinephrine reuptake inhibitor, *MAOI* monoamine oxidase inhibitors

### Correlation with metabolism and perfusion

No significant SPM(T) results were found for metabolism in correlation with either the number of depressive episodes or the depression duration, even for a less stringent threshold at an uncorrected p-voxel < 0.005.

On the other hand, significant correlations were found with perfusion at p-voxel < 0.001 and *k* > 20. The increased number of depressive episodes was correlated with decreased perfusion of the right middle frontal cortex (T-voxel max = 5.50, *k* = 106), the right anterior cingulum cortex (ACC) (T-voxel max = 5.13, *k* = 75), the right medial temporal cortex (T-max = 4.93, *k* = 79), the left precuneus (T-voxel max = 4.80, *k* = 66) and the right insula (T-voxel max = 4.52, *k* = 61). The increased depression duration was correlated with the perfusion of the right ACC (T-voxel max = 5.31, *k* = 58).

Spearman’s correlations on extracted clusters confirmed a negative correlation between the number of depressive episodes and perfusion of the right medial temporal cortex (rho = − 0.84, *p* = 0.00002), the left precuneus (rho = − 0.50, *p* = 0.02), the right ACC (rho = − 0.49, *p* = 0.02) and the right insula (rho = − 0.58, *p* = 0.01). Negative correlations were also confirmed between depression duration and perfusion of the right ACC (rho = − 0.61, *p* = 0.01). These same clusters were extracted for brain glucose metabolism without significant correlation.

**Fig. 1:**
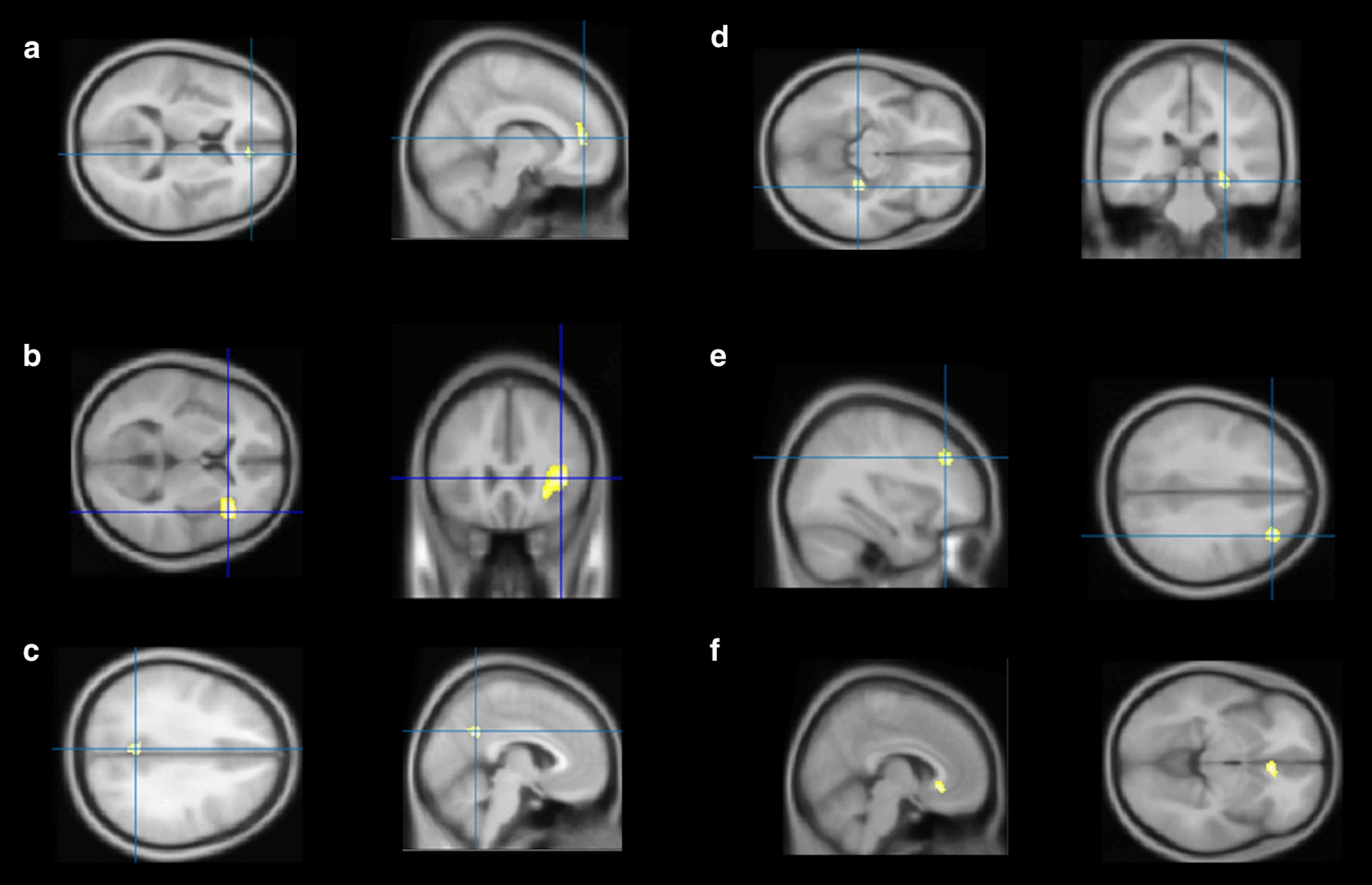
Anatomical localization of significant perfusion SPECT findings (p-voxel < 0.001 uncorrected, *k* > 20). The increased number of depressive episodes is correlated with decreased perfusion of: **a** the right anterior cingulate cortex (ACC) (T-voxel max = 3.85, *k* = 75), **b** the right insula (T-voxel max = 3.85, *k* = 26), **c** the left precuneus (T-voxel max = 3.85, *k* = 62), **d** the right medial temporal cortex (T-max = 3.85, *k* = 66) and **e** the right middle frontal cortex (T-voxel max = 3.85, *k* = 106). **f** The increased depression duration is correlated with decreased perfusion of the right ACC (T-voxel max = 5.31, *k* = 236)

**Fig. 2:**
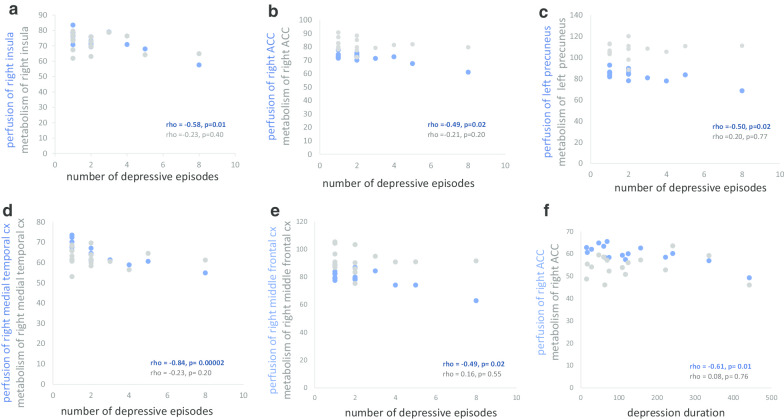
Scatter plot of Spearman’s correlations on extracted clusters. The increased number of depressive episodes is correlated with decreased perfusion of: **a** the right insula (rho = − 0.58, *p* = 0.01), **b** the right anterior cingulate cortex (ACC) (rho = − 0.49, *p* = 0.02), **c** the left precuneus (rho = − 0.50, *p* = 0.02), **d** the right medial temporal cortex (rho = − 0.84, *p* = 0.00002) and **e** the right middle frontal cortex (rho = − 0.49, *p* = 0.02). This correlation is not significant with the metabolism of: **a** the right insula (rho = − 0.23, *p* = 0.40), **b** the right ACC (rho = − 0.21, *p* = 0.20), **c** the left precuneus (rho = 0.20, *p* = 0.77), **d** the right medial temporal cortex (rho = − 0.23, *p* = 0.20) and **e** the right middle frontal cortex (rho = 0.16, *p* = 0.55). **f** The increased depression duration is correlated with decreased perfusion of the right ACC (rho = − 0.61, *p* = 0.01). This correlation is not significant with the metabolism of the right ACC (rho = 0.08, *p* = 0.76)

These results are presented in Figs. 1 and 2.

## Discussion

We conducted a retrospective study in a sample of 16 patients suffering from TRD who all underwent a cerebral perfusion SPECT with ^99m^Tc-HMPAO and a metabolic PET with ^18^F-FDG. No relevant clinical changes were found upon evaluation at these two time points, especially for disease characteristics and treatments. Whole-brain voxel-based analysis revealed distinct results between perfusion and glucose metabolism; this could, at least partly, explain the previous variability of findings in the literature for these two biomarkers [7]. Significant negative correlations were found between number of episodes and perfusion of the right middle frontal cortex, the right ACC, the right insula, the right medial temporal cortex and the left precuneus, as well as between the depression duration and right ACC perfusion, while no relationship was obtained for glucose metabolism.

Number of episodes and illness duration are considered as risk factors of pharmaco-resistance and are involved in depression recurrence [11, 12]. They also may signal risk of residual symptoms, such as sleep disturbances, executive impairments and anxiety [13]. They are considered by a group of experts as great indicators of prognosis and severity of TRD [14–16]. Identifying their relationship with cerebral functioning could improve resistance management and selection of potential cerebral therapeutic targets. Correlations with these two variables have already been described in a few previous neuroimaging studies. A voxel-based study of 127 subjects suffering from TRD showed a weak negative correlation between the duration of illness and brain ^99m^Tc-ECD SPECT perfusion in bilateral cingulate and orbital cortices [17]. A recent MRI meta-analysis including morphometry studies found significant grey matter reduction in the rostral part of the anterior cingulum related to illness duration and repeated depressive episodes [18]. Moreover, our findings are concordant with another ^18^F-FDG PET study. In 18 hospitalized patients with unipolar depression, Mayberg et al. [19] revealed no significant relationship between brain metabolism and illness chronicity.

The brain regions revealed in this study are known to be involved in TRD [7, 20]. The insula is particularly involved in emotional identification and in the affective state in response to a stimulus. Resting-state hyperactivity of the insula has been linked, in MDD, to pathological self-focused mental ruminative behaviours [21]. On the other hand, the ACC modulates the link between ventral and dorsal networks involved in regulation of emotion. The dorsal ACC is specifically implicated in executive functions through the cognitive control network, and its subgenual subdivision is focused on emotional experience and processing [22]. Furthermore, the insula and ACC both belong to the salience network, which participates in judgement alteration and negative thoughts in MDD [23]. They also both interact with the default mode network and contribute to the alteration of attentional system and to the introspection in MDD [24]. The precuneus, medial frontal and medial temporal cortices have been mainly implicated through a default mode network and could also contribute to low self-esteem and ruminations [25]. The number of episodes and depression duration seem to alter brain perfusion, especially in cerebral areas linked to emotional and cognitive symptoms of depression. It could suggest their key role in pharmaco-resistance and recurrence by enhancing the cortico-limbic dysregulation described in unipolar depression.

PET is one of the main neuroimaging techniques evaluated in recent psychiatry research [20]. Indeed, PET is usually preferred to SPECT because of better spatial resolution. Cerebral glucose metabolism and perfusion have been considered as coupled for a long time, because the brain consumes approximately 20% of total body oxygen and 25% of total body glucose. The most important energy source for the brain is adenosine triphosphate (ATP), which is produced almost entirely by the oxidative metabolism of glucose [26]. However, their consistent correlations are presently questioned and in part justified by other mechanisms of blood flow regulation, which could produce a different cartography of cerebral perfusion from the metabolic one. This has already been highlighted in healthy subjects [27]. Furthermore, uncoupling between glucose and oxygen metabolism, via oxygen depletion and induction of downstream hypoxia response pathways, could play a key role in neurodegenerative diseases [28]. The underlying mechanisms of drug resistance in depression remain misunderstood in the current literature [29]. Nevertheless, it is not excluded that psychiatric disease could involve the same uncoupling mechanisms. Functional compensation, more specifically involving brain metabolism, may also occur. Further fundamental studies are necessary to specify the contribution of these mechanisms in depression.

The main limitations of the study were a retrospective design and a small sample size. Our results concerned patients with TRD, and they are not generalizable to all patients suffering from MDD. Subjects were mostly women with a mean age above 50 years old, which are expected sociodemographic characteristics; advanced age and female gender are risk factors for TRD [30, 31]. Moreover, the interval delay between the two acquisitions was significant and could impact the findings. This difference was expected because of the sequence of exploration. Indeed, the order was fixed (PET after SPECT), with inevitably a difference using a rank test. Nevertheless, the number of depression episodes is strictly the same at the two times of the study, and the difference between depression duration was weak among the groups (136.44 ± 120 months vs. 132.56 ± 121.48 months). Treatments and modifications of therapy between PET and SPECT explorations could have also impacted our findings, possibly more the metabolism than the perfusion. Nevertheless, these changes were limited and statistically non-significant. In detail, a selective serotonin reuptake inhibitor was changed for two patients, including one for a serotonin–norepinephrine reuptake inhibitor, and one antidepressant was potentialized by an antipsychotic during the interval delay, which constitute common changes in the therapeutic strategy of depression. Moreover, no treatment interruption occurred between the two examinations. To our knowledge, this is nevertheless the first study that focuses on a single group of patients to compare perfusion and metabolism in MDD. Finally, the perfusion and metabolic measurements were semiquantitative, as used in clinical practice. Further studies with absolute quantification could better explain the differences observed in this preliminary report.

## Conclusions

This preliminary study demonstrates that the clinical characteristics of TRD are more significantly associated with brain perfusion than with glucose metabolism, highlighting the value of brain SPECT despite its less favourable spatial resolution and image quality compared to PET. These findings warrant other comparative studies between these two imaging techniques to provide a more frequent use of brain perfusion in future psychiatric research.

## Data Availability

The data that support the findings of this study are available from the corresponding author upon reasonable request.
